# External validation of the feeding intolerance prediction model (NOFI) in critically ill patients: A post hoc analysis of a large-scale randomized controlled trial

**DOI:** 10.1016/j.jointm.2025.12.004

**Published:** 2026-02-02

**Authors:** Youquan Wang, Yanhua Li, Hongxiang Li, Nan Li, Xinyu Li, Dongfang Lv, Lingling Bao, Xuewen Feng, Lu Ke, Dong Zhang

**Affiliations:** 1 Department of Critical Care Medicine, The First Hospital of Jilin University, Changchun, Jilin, China; 2 Department of Critical Care Medicine, Jinling Hospital, Medical School of Nanjing University, Nanjing, Jiangsu, China; 3 National Institute of Healthcare Data Science, Nanjing University, Nanjing, Jiangsu, China; 4 Chinese Critical Care Nutrition Trials Group (CCCNTG), No. 22 Hankou Road, Nanjing, Jiangsu, China

**Keywords:** Feeding intolerance, Nomogram, Intensive care unit, Clinical outcomes, External validation

## Abstract

**Background:**

Feeding intolerance (FI) is associated with poor prognosis of critically ill patients. This study aims to externally validate the performance of a FI model (NOFI), which was developed using primary diagnosis, Acute Physiology and Chronic Health Evaluation II (APACHE II) score, and acute gastrointestinal injury (AGI) grade to predict FI occurrence in critically ill patients.

**Methods:**

The NOFI model was developed using a retrospective cohort and was preliminarily validated in a prospective study. A post hoc analysis was then conducted using data from a multi-center randomized controlled trial to select patients who initiated early enteral nutrition as an external validation dataset. The predictive performance of NOFI was evaluated in terms of discrimination, calibration, and clinical utility. FI risk stratification was defined as follows: The NOFI prediction probability >0.85 (high risk), 0.30–0.85 (middle risk), or <0.30 (low risk). Multivariate Cox regression was performed to explore the association between FI risk and 28-day mortality.

**Results:**

Among the 1545 patients included, 856 developed FI. The area under the receiver operating characteristic curve (AUROC) for NOFI was 0.723 (95% confidence interval [CI]: 0.697 to 0.748). The prediction model was able to accurately predict FI, with a sensitivity of 75.8%, specificity of 61.1%, positive predictive value of 70.8%, and negative predictive value of 67.0%. FI risk stratification based on NOFI was associated with 28-day mortality (*P*=0.003), with the high-risk FI group having a higher 28-day mortality compared to the low-risk group, with a hazard ratio (HR) of 2.28 (95% CI: 1.36 to 3.82). The high-risk FI group also had a higher 28-day mortality compared to the middle-risk group (HR=1.54, 95% CI: 1.11 to 2.12). However, no significant difference in 28-day mortality was found between the middle-risk and low-risk FI groups (HR=1.48, 95% CI: 0.93 to 2.37).

**Conclusions:**

NOFI demonstrated reasonable performance in the prediction of FI. Patients at high risk for FI as stratified by the NOFI had a higher risk of 28-day mortality.

## Introduction

For critically ill patients who are unable to eat orally, guidelines recommend early enteral nutrition (EEN) rather than parenteral nutrition (PN) or delayed nutritional therapy.^[^[1], [2], [3]^]^ However, in critically ill patients, gastrointestinal (GI) dysfunction may occur to varying degrees, which may hinder the effective implementation of enteral nutrition (EN).^[^^4^^]^ When the intensity of EN does not align with the GI function, feeding intolerance (FI) may arise, potentially leading to a reduction or even suspension of EN. Furthermore, FI may also be associated with poorer clinical outcomes.^[^^5^^]^

The European Society of Intensive Care Medicine (ESICM) guidelines were the first to propose a definition of FI, which involves a comprehensive assessment based on GI symptoms and the feeding dosage initiated within 72 h of EN administration.^[^^4^^]^ However, the assessment of FI typically takes several days, which hinders the possibility of early nutritional intervention. Individualized prediction of the likelihood of FI in critically ill patients, followed by the development of tailored nutritional plans, may reduce the incidence of FI.^[^^6^^]^ In recent years, many predictive models for FI in critically ill patients have been developed.^[^[6], [7], [8], [9], [10]^]^ However, these models differ in terms of the population they apply to, the diagnostic criteria for FI, and the diagnostic time window, which makes it difficult to compare them. Only a few studies have published the coefficients of the models, which has hindered the external validation of FI prediction models.

Given these limitations, we chose to use data from a multicenter, cluster-randomized controlled trial in China to independently validate the FI prediction model (NOFI) and determine whether this model is of potential clinical value. This model was developed using retrospective data and has been preliminarily validated in prospective studies, including three predictive factors: primary diagnosis, Acute Physiology and Chronic Health Evaluation II (APACHE II) score, and acute gastrointestinal injury (AGI) grade. There are three main reasons for selecting this model for validation: (1) The FI definition used in the model was proposed by the 2012 ESICM^[^^4^^]^ and includes two dimensions of assessment – GI symptoms and feeding volume – which is the most widely used definition in clinical practice; (2) The model assesses FI within the first 7 days of ICU admission, corresponding to the acute phase of critical illness, which is the period most relevant to clinical decision-making. Other FI prediction models use heterogeneous assessment windows that are sometimes longer or shorter, limiting their comparability and clinical utility; and (3) Unlike most other FI prediction models, which lack external validation, this model has already demonstrated promising results in a preliminary independent external validation cohort, making it a more reliable candidate for further testing. We will evaluate NOFI from the perspectives of discrimination, calibration, and clinical utility, assuming that the performance of this model can reach a middle or higher level and can be used in clinical practice.

## Methods

### Study design and patients

This study presents a secondary analysis of the multicenter, cluster-randomized controlled trial (NEED) .^[^^11^^]^ In summary, 97 intensive care units (ICUs) across China were randomly assigned to either a guideline group that implemented a feeding protocol or a control group that followed local clinical practices. The feeding guidelines outline the appropriate timing for initiating EN and PN, criteria for assessing FI, and methods for adjusting the feeding rate based on intolerance evaluations to meet the nutritional goals of 25–30 kcal/(kg·day) for calorie and 1.2–2.0 g/(kg·day) for protein. A total of 2772 patients were enrolled within 24 h of ICU admission from 90 ICUs between March 26, 2018, and July 4, 2019.

The original study protocol received approval from the Ethics Committee of Jinlin Hospital (No. 22017NZKY-019-02), and written informed consent was obtained from all participants or their legally authorized representatives prior to enrollment. The trial was registered in the ISRCTN registry (https://www.isrctn.com/ISRCTN12233792; registration date: March 22, 2018).

Our study included an analysis of a subset of participants based on specific inclusion criteria: (1) initiation of EN within 48 h of ICU admission; and (2) patients with at least 3 days of enteral feeding. We excluded patients classified as AGI grade IV, as they were unable to receive EN in the short term. Additionally, we excluded patients who were receiving oral intake, as uncertainty about energy intake could lead to undetermined FI.

### Prediction model of feeding intolerance-NOFI

NOFI is a prediction model based on a nomogram to predict FI in critically ill patients during the acute phase of critical illness (within 7 days), with a focus on EEN initiation within 48 h. The predictors include the primary diagnosis, APACHE II score, and AGI grade. The model’s calculation formula can be found in Supplementary Table S1. NOFI was developed using a retrospective cohort and was preliminarily validated in a prospective study.

The online nomogram/calculator was developed using the Shiny application; The tool can be obtained at the following URL: youquan.shinyapps.io/shiny_app/.

The information on all the FI prediction models we know is summarized in Supplementary Table S2.

### Data collection

All data were obtained from electronic databases of the NEED trial, including patients’ general clinical information (such as age, sex, body mass index, and number of comorbidities), primary diagnosis, APACHE II score, Sequential Organ Failure Assessment (SOFA) score, AGI grade, modified nutrition risk in critically ill (mNUTRIC) score, specific details of nutritional implementation (such as the timing of EN initiation, formula used, actual daily feeding volume, and feeding route), as well as GI symptoms and adverse events. For non-obese patients, the actual weight recorded on the day of admission was used to calculate the average daily energy and protein intake. For obese patients (body mass index [BMI] ≥30 kg/m^2^), the ideal body weight was used, calculated using the Broca index: height (cm)−100 (for males) or height (cm)−105 (for females).

### Assessment of FI

In this study, FI was diagnosed according to the criteria outlined in the 2012 ESICM guidelines. Specifically, FI was defined as (1) GI symptoms (vomiting, a high gastric residual volume [single residual volume greater than 200 mL] or diarrhea [>3 loose or liquid stools per day, stool weight exceeding 200–250 g/day or 250 mL/day]) and discontinuation or reduction of EN; and/or (2) 20 kcal/(kg·day) not reached after 72 h of EN.

Two investigators evaluated the 72-h period after initiation of enteral nutrition (EN) and classified EN as present or absent based on the following criteria: (1) presence of any gastrointestinal symptoms; (2) failure to achieve 20 kcal/(kg·day) within 72 h of EN; or (3) both. They evaluated FI separately and finally integrated the FI results. When the FI evaluation results are inconsistent, HX Li and D Zhang discuss and determine the final FI evaluation results.

The assessors’ results on whether these patients developed FI were consistent because the data on GI symptoms and daily nutritional intake in the NEED dataset were inherent.

### Statistical analyses

The normality of continuous variables was assessed using the Shapiro–Wilk test, and homogeneity of variance was evaluated using Levene’s test. For normally distributed variables with equal variances, Student’s *t*-test was used; if variances were unequal, Welch’s *t*-test was applied. For variables with skewed distributions, the Wilcoxon–Mann–Whitney *U* test was used, and results were reported as the median and interquartile range (IQR). Categorical variables were described by frequency (percentage) and compared using either Pearson’s Chi-squared test or Fisher’s exact test, depending on which was appropriate.

An independent external validation of a clinical prediction model was performed to assess its discriminative ability. The area under the receiver operating characteristic (ROC) curve (AUROC) was used to evaluate the model’s performance. The best-fitting model and nomogram were validated and calibrated using bootstrapping techniques. A total of 1000 resamples were generated using the bootstrap method, and the resulting bootstrap-corrected AUROC, along with its 95% confidence interval (CI) were reported. The calibration of the nomogram was further evaluated using the Hosmer–Lemeshow test.

In addition, various performance metrics of the prediction model were calculated, including sensitivity, specificity, positive predictive value (PPV), and negative predictive value (NPV). Decision curve analysis (DCA) and clinical impact curve (CIC) were applied to assess the clinical utility of the nomogram.

In the DCA of NOFI, we obtained two truncated values (0.30 and 0.85) based on whether the difference in net returns was greater than 0.05.^[^^6^^]^ Two cut-off values (0.30 and 0.85) were identified, showing that the use of NOFI would provide greater clinical benefit when the threshold probability for the occurrence of FI was between 0.30 and 0.85, compared to assuming FI would either always occur or never occur. Based on these cut-off values, patients were categorized into low, middle, and high-risk groups for FI occurrence. A multivariate Cox regression analysis was performed to assess the association between the risk of FI and 28-day mortality, adjusting for potential confounders including APACHE Ⅱ score, AGI grade, and mechanical ventilation (MV), which are factors that may be associated with mortality in AGI patients.

Statistical analysis was conducted in JMP (SAS Institute, Cary, NC, USA) and R version 4.3.1 (R Foundation for Statistical Computing, Vienna, Austria) with RStudio version 1.0.136 (RStudio Inc, Boston, MA, USA).

## Results

### Patients baseline characteristics

**Figure 1:**
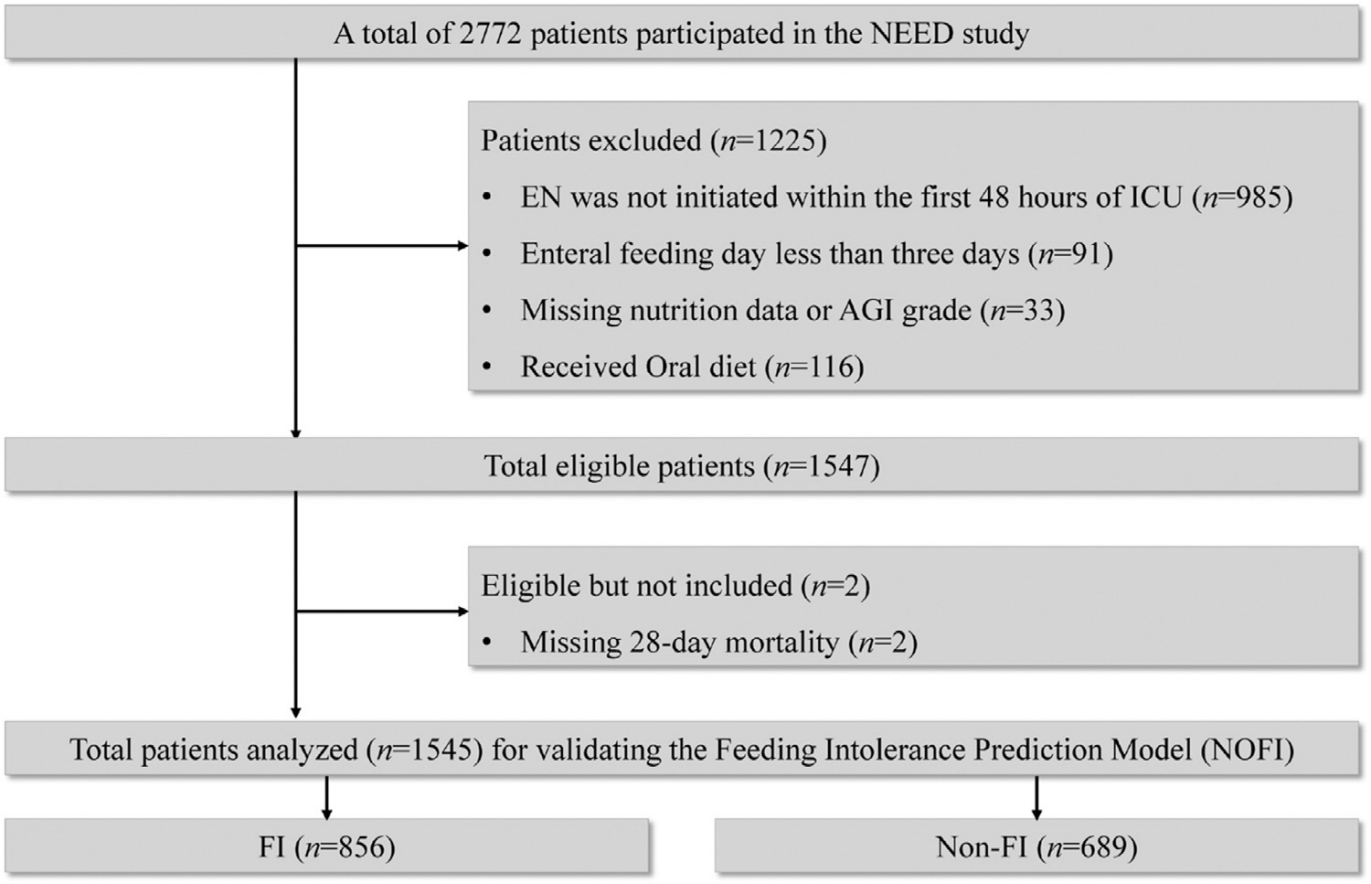
Flowchart of patient selection. AGI: Acute gastrointestinal injury; FI: Feeding intolerance; ICU: Intensive care unit; NOFI: Nomogram of feeding intolerance.

Out of the 2772 patients enrolled in the original trial, 1545 were eventually included in the analysis (Figure 1). The mean age of the patients was (62.2±17.6) years, and their mean BMI was (22.7 ± 3.2)kg/m^2^. Among them, 67.1% were male. The median APACHE Ⅱ score was 18.0 (IQR: 14.0–22.0), the median SOFA score was 7.0 (IQR: 5.0–9.0), and the median mNUTRIC score was 4.0 (IQR: 3.0–6.0). During the first 7 days after ICU admission, 856 patients experienced FI, while 689 patients did not. The baseline characteristics of the research objects are summarized in Table 1.

**Table 1:** Baseline characteristics of patients.

Variables	Total (*n*=1545)	FI (*n*=856)	Non-FI (*n*=689)	*P* value
Age(years)	62.2 ± 17.6	62.4 ± 17.5	62.0 ± 17.8	0.656
Male	1036 (67.1)	610 (71.3)	426 (61.8)	<0.001
BMI (kg/m^2^)	22.7±3.2	23.2±3.2	22.1±3.2	<0.001
BMI range (kg/m^2^)				<0.001
<18.5	91 (5.9)	23 (2.7)	68 (9.9)	
18.5–25.0	1160 (75.1)	647 (75.6)	513 (74.5)	
25.0–30.0	255 (16.5)	157 (18.3)	98 (14.2)	
>30.0	39 (2.5)	29 (3.4)	10 (1.5)	
Primary diagnosis				0.045
Neurologic	266 (17.2)	147 (17.2)	119 (17.3)	
Cardiovascular	374 (24.2)	226 (26.4)	148 (21.5)	
Respiratory	729 (47.2)	377 (44.0)	352 (51.1)	
Multi trauma	32 (2.1)	18 (2.1)	14 (2.0)	
Others	144 (9.3)	88 (10.3)	56 (8.1)	
Critical care related score*				
APACHE II	18.0 (14.0–22.0)	18.0 (15.0–23.0)	18.0 (13.0–22.0)	<0.001
SOFA	7.0 (5.0–9.0)	7.0 (5.0–10.0)	7.0 (5.0–9.0)	0.005
AGI grade				<0.001
≤Ⅰ	1228 (79.5)	608 (71.0)	620 (90.0)	
Ⅱ	265 (17.2)	202 (23.6)	63 (9.1)	
Ⅲ	52 (3.4)	46 (5.4)	6 (0.9)	
mNUTRIC	4.0 (3.0–6.0)	4.0 (3.0–6.0)	4.0 (3.0–5.0)	0.011
Number of co-morbidities	2 (1–3)	2 (1–3)	2 (1–3)	0.472
Organ support therapy				
MV†	939 (60.8)	536 (62.6)	403 (58.5)	0.099
CRRT†	184 (11.9)	115 (13.4)	69 (10.0)	0.039
Vasopressors*	396 (25.6)	242 (28.3)	154 (22.4)	0.008
Source of ICU admission				0.741
Surgical	392 (25.4)	220 (25.7)	172 (25.0)	
Medical	1153 (74.6)	636 (74.3)	517 (75.0)	
EN delivery within 7 days after enrollment				
Mean daily EN energy intake (kcal/(kg·day) )	15.8 (11.5–20.2)	12.3 (8.8–15.2)	20.6 (18.1–23.8)	<0.001
Mean daily EN protein intake (g/(kg·day) )	0.63 (0.46–0.81)	0.49 (0.35–0.61)	0.82 (0.72–0.95)	<0.001
Study interventions				0.389
NEED group	867 (56.1)	472 (55.1)	395 (57.3)	
Control group	678 (43.9)	384 (44.9)	294 (42.7)	
Tube feeding route				0.986
Nasogastric	1507 (97.5)	835 (97.5)	672 (97.5)	
Postpyloric	38 (2.5)	21 (2.5)	17 (2.5)	
Results of the first 24 h of testing				
Albumin (g/L)	31.6 (27.8–36.1)	31.2 (27.5–36.0)	32.0 (28.0–36.2)	0.164
Pre-albumin (g/L)	0.15 (0.11–0.22)	0.15 (0.11–0.21)	0.16 (0.11–0.23)	0.314
C-reactive protein (mg/L)	54.5 (16.9–114.0)	57.0 (16.0–119.0)	50.0 (18.0–108.4)	0.252
Lac (mmol/L)	1.8 (1.2–2.7)	1.8 (1.2–2.8)	1.7 (1.1–2.7)	0.403

Data were presented as mean ± standard deviation, median (interquartile range) or *n* (%).

⁎ Within 24 h of ICU admission;

† Within 7 days of ICU admission. APACHE II: Acute physiology and chronic health evaluation II; AGI: Acute gastrointestinal injury; BMI: Body mass index; CRRT: Continuous renal replacement therapy; EN: Enteral nutrition; FI: Feeding intolerance; ICU: intensive care unit; mNUTRIC: Modified nutrition risk in critically ill score; MV: Mechanical ventilation; SOFA: Sequential organ failure assessment.

### Results of FI assessment

Among the 856 patients who developed FI, 38 patients were diagnosed based solely on GI symptoms, 79 patients could not reach at least 20 kcal/(kg·day) for body weight (BW) via enteral route within 72 h of feeding attempt, and 739 patients met the diagnostic criteria for FI based on both GI symptoms and enteral feeding doses.

**Figure 2:**
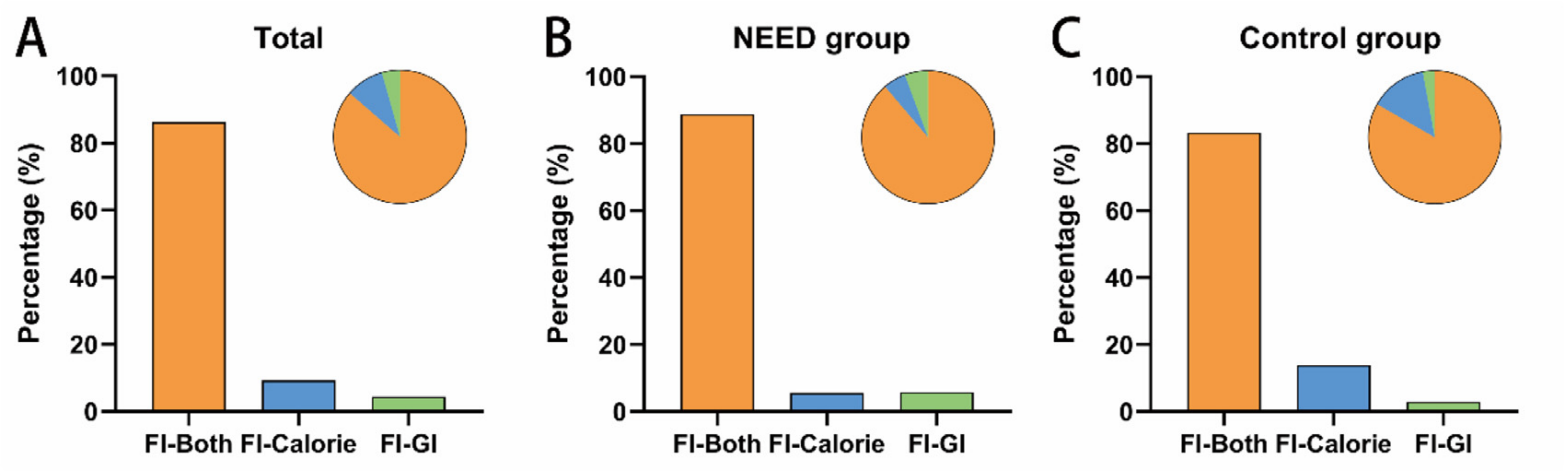
The diagnostic process and proportion of FI in the total data (A), the NEED group (B), and the control group (C). FI-Both: Both GI symptoms and enteral feeding doses met the diagnostic criteria for FI; FI-Calorie: at least 20 kcal/(kg·day) for body weight via enteral route cannot be reached within 72 h of feeding attempt, but GI symptoms were not sufficient to diagnose FI; FI-GI: FI was diagnosed based solely on GI symptoms. FI: Feeding intolerance; GI: Gastrointestinal.

The diagnostic process and proportion of FI in the total data, the trial assignment and the control group are shown in Figure 2.

### Patients EN intake characteristics

The median daily EN energy intake for all patients during the 7-day ICU stay was 964.3 (IQR: 714.3–1242.9) kcal/day; the median daily EN energy intake of patients with FI was 785.7 (IQR: 535.7–959.3) kcal/day during 7 days in ICU and the median daily EN energy intake of patients without FI was 1232.1 (IQR: 1057.1–1428.6) kcal/day during the 7 days in ICU. Daily nutritional calorie intake for all patients, FI patients, and non-FI patients during the 1–7 days after ICU admission was shown in Supplementary Figure S1. Daily nutritional calorie intake for FI patients and non-FI patients during the 1–7 days after ICU admission in the NEED and control subgroups is shown in Supplementary Figure S2.

### Validation of the discrimination, calibration, and clinical utility of the NOFI model

#### Discrimination and calibration

**Figure 3:**
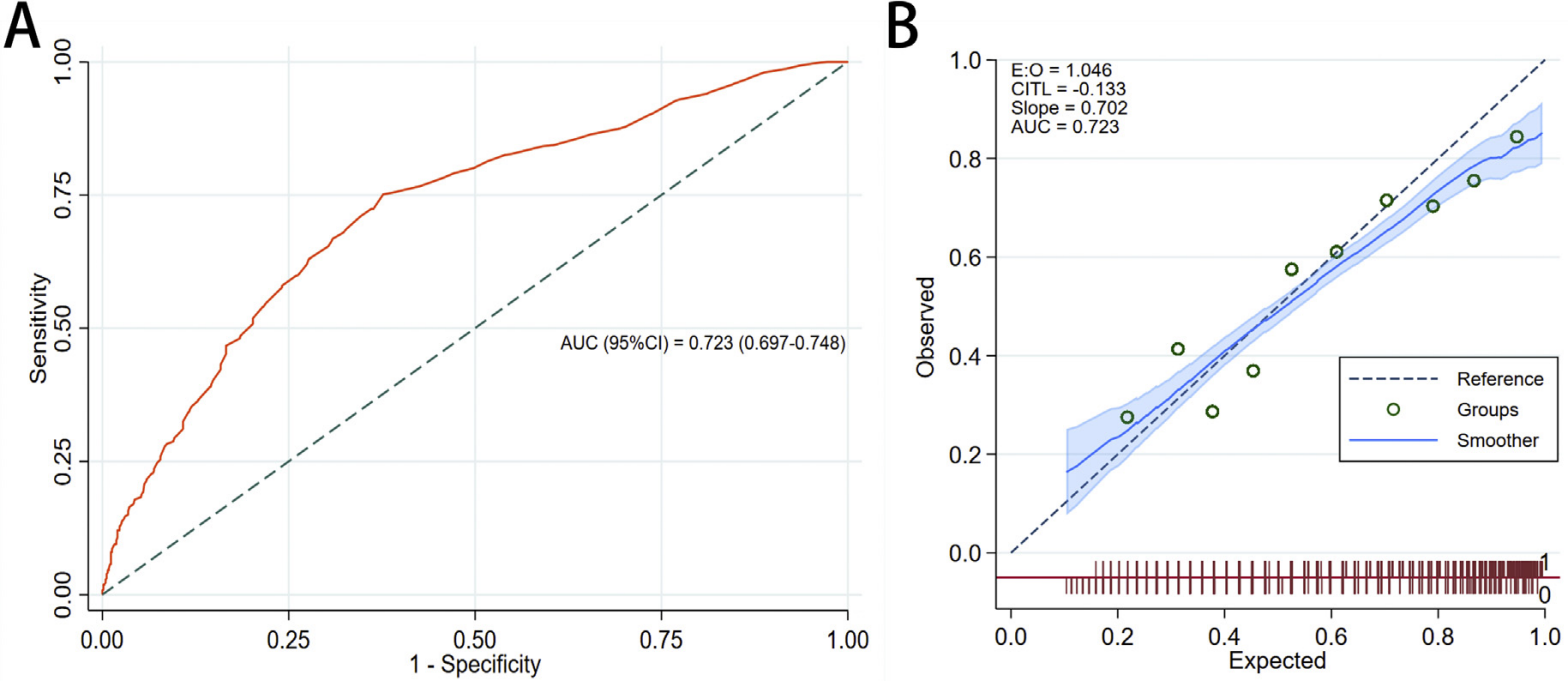
The discrimination (A) and calibration (B) of NOFI. AUC: Area under the curve; CI: Confidence interval; NOFI: Nomogram of feeding intolerance.

The AUROC for NOFI is 0.723 (95% CI: 0.697 to 0.748). This indicates that the predictive model had a fair ability to discriminate between patients who developed FI and those who did not (Figure 3A).

The calibration curve demonstrated a reasonable agreement between predicted and observed probabilities of FI, as shown by its proximity to the 45° reference line (Figure 3B). The slope of 0.702 suggests that the model slightly underestimates the risk of FI, while the intercept (E:0=1.046) indicates only a small systematic bias. Together, these results suggest that although the model slightly underpredicts FI, its calibration is acceptable for clinical use. This means that in real-world settings, clinicians can generally trust the predicted probabilities from the model but should remain aware of a tendency toward slight underestimation, particularly in higher-risk patients.

**Figure 4:**
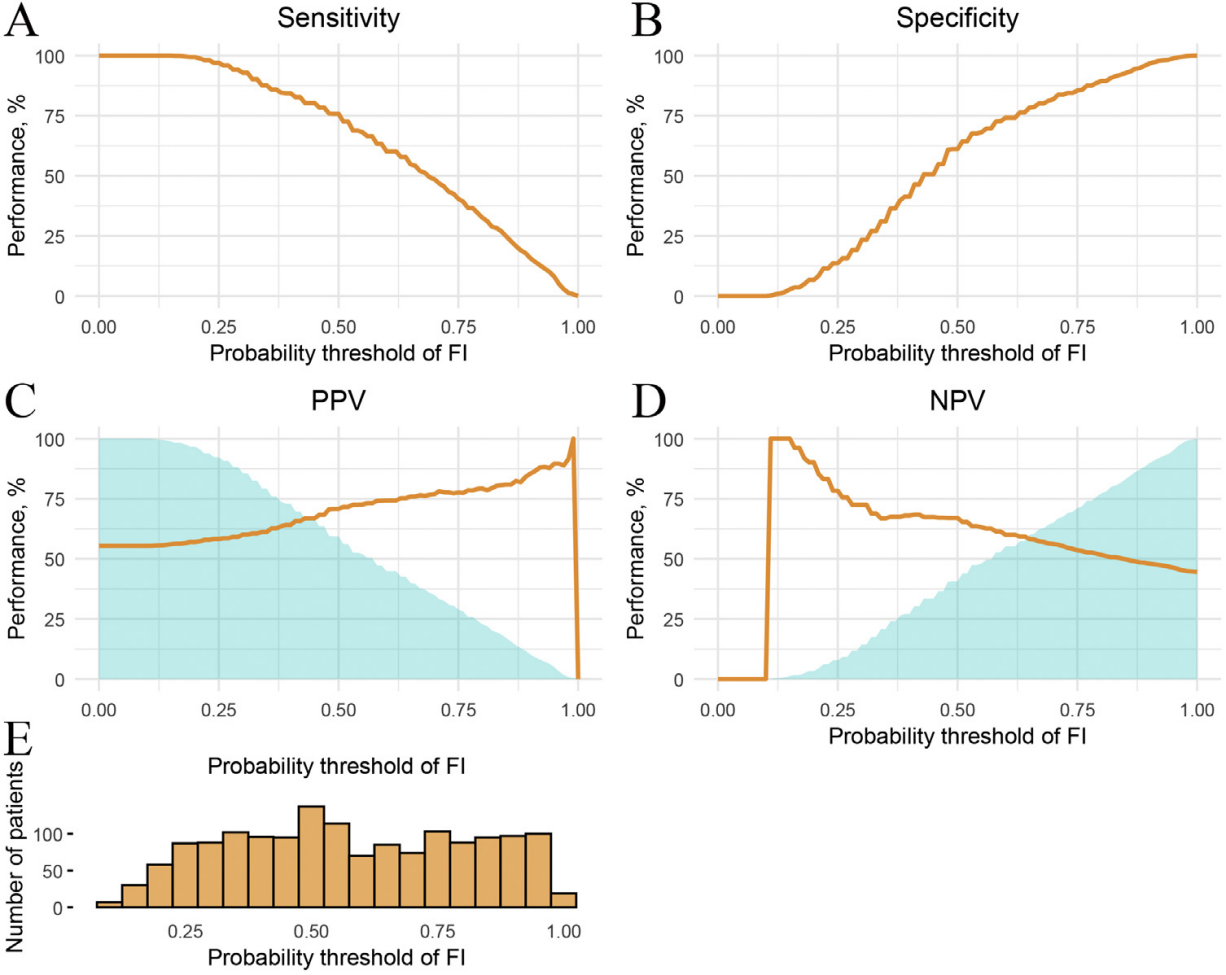
Threshold performance graphs of NOFI. A: Sensitivity plot: the curve shows sensitivity across different probability thresholds used to define a positive classification. B: Specificity plot: the curve shows specificity across different probability thresholds used to define a positive classification. C: PPV plot, the blue-shaded region refers to the percentage of patients classified as positive. D: NPV plot, the blue-shaded region refers to the percentage of patients classified as negative. E: Distribution of predicted FI probabilities across the study population (*y*-axis represents patient count). FI: Feeding intolerance; NOFI: Nomogram of feeding intolerance; NPV: Negative predictive value; PPV: Positive predictive value.

At the optimal threshold of 0.49, the prediction model achieved a sensitivity of 75.8% (95% CI: 72.8 to78.6%) and a specificity of 61.1% (95% CI: 57.4 to 64.7%), indicating a relatively strong ability to identify true positive cases while maintaining moderate discrimination of negative cases. The PPV was 70.8% (95% CI: 67.7 to 73.6%), suggesting that a high proportion of patients identified as high risk truly experienced the outcome. The NPV was 67.0% (95% CI: 63.3 to 70.6%), reflecting a moderate likelihood that patients predicted to be at low risk did not experience the event. In addition, the F1 score, which represents the harmonic mean of sensitivity and positive predictive value, was 0.73, indicating a balanced performance in correctly identifying patients with FI while limiting false positives. Overall, the model demonstrated reasonable predictive performance, particularly in its capacity to detect true cases (Figure 4).

#### Clinical utility

**Figure 5:**
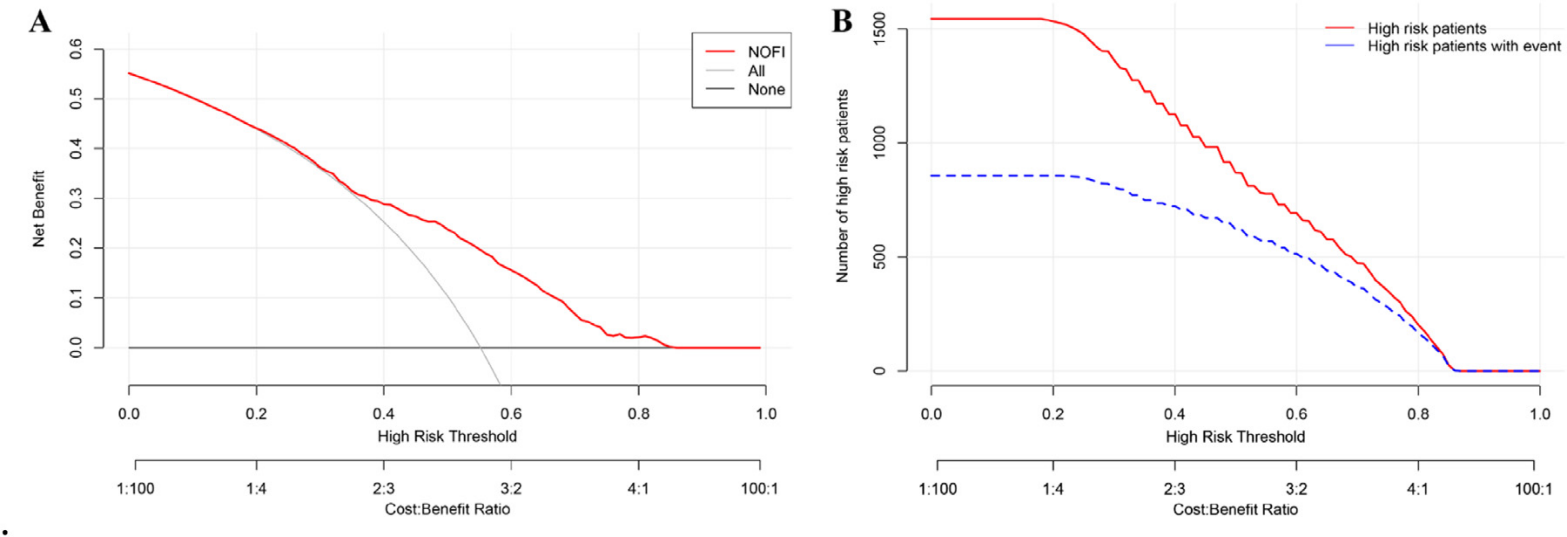
The results of (A) the decision curve analysis and (B) the clinical impact curve of the NOFI. NOFI: Nomogram of feeding intolerance.

The decision curve analysis (Figure 5A) demonstrated that the NOFI yielded a greater net benefit than either the “treat-all” or “treat-none” strategies across a wide range of threshold probabilities (approximately 0.05–0.80), with the greatest clinical utility observed between thresholds of 0.1 and 0.6. This suggests that the model has practical value in guiding treatment decisions by improving patient selection.

The clinical impact curve (Figure 5B) further supported the model’s utility, showing that the number of patients classified as high risk was reasonably aligned with the number of true positive cases within the same threshold range. This indicates that the model can effectively reduce overtreatment while still capturing a substantial proportion of true events, especially at clinically relevant thresholds.

### Association between FI risk and 28-day mortality

**Figure 6:**
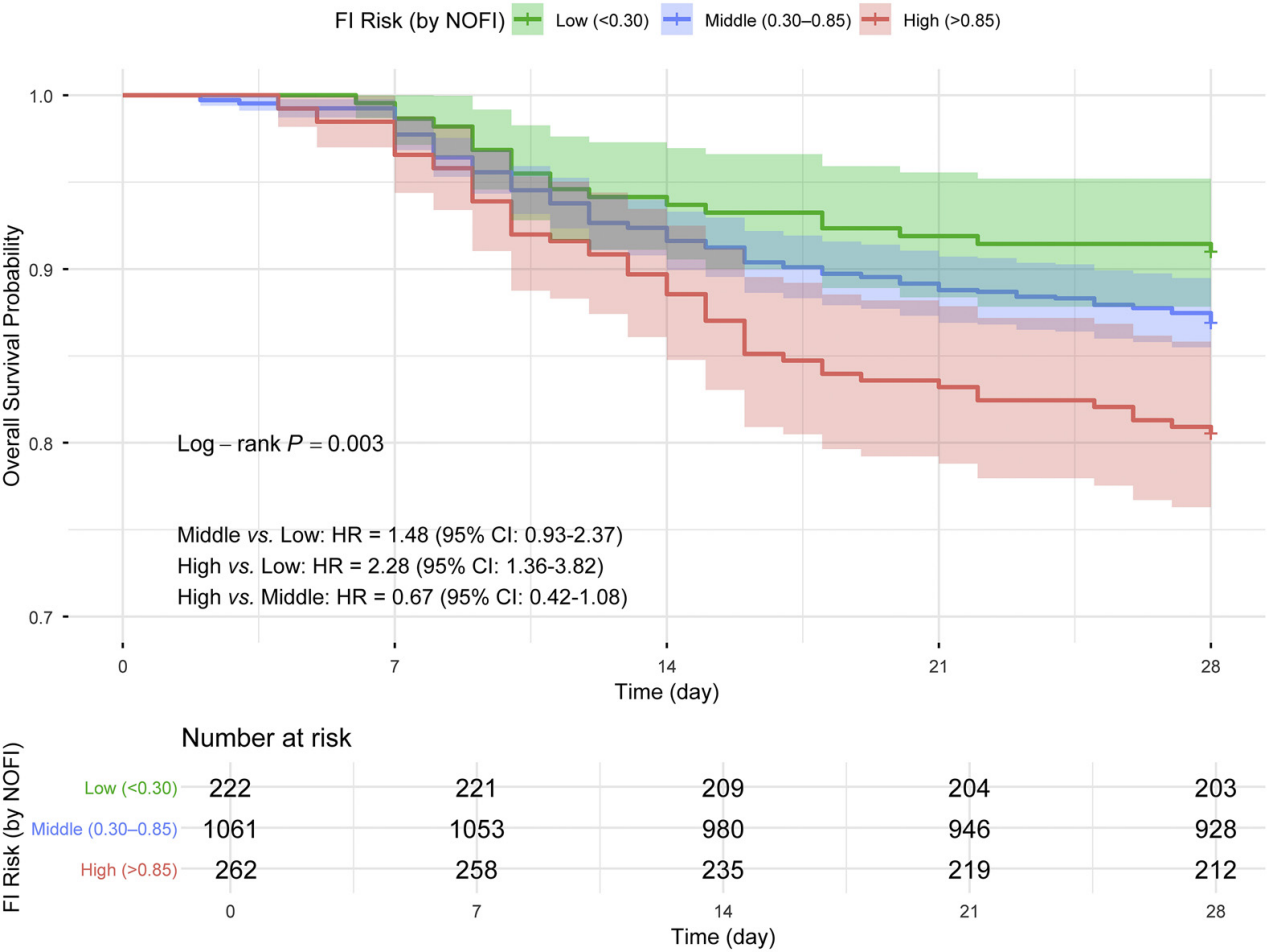
Cox regression survival curves between high, middle, and low risk of FI occurrence and 28-day mortality. Cox regression adjusted for APACHE II score, AGI grade, and MV. Time zero (t₀) was defined as intensive care unit admission (day 0). APACHE II: Acute physiology and chronic health evaluation II; AGI: Acute gastrointestinal injury; CI: Confidence interval; HR: Hazard ratio; MV: Mechanical ventilation; NOFI: Nomogram of feeding intolerance.

A total of 222 patients were assessed as low risk for FI, 1061 patients were assessed as middle risk for FI, and 262 patients were assessed as high risk for FI. A multivariate Cox regression analysis was conducted to examine the association between FI risk stratification and 28-day mortality, adjusted for APACHE Ⅱ score, AGI grade, and MV. The results showed that the FI risk stratification based on NOFI was associated with 28-day mortality (*P*=0.003). Among them, the high-risk FI group had a higher 28-day mortality compared to the low-risk FI group, with hazard ratio (HR)=2.28 (95% CI: 1.36 to 3.82). The high-risk FI group also had a higher 28-day mortality compared to the middle-risk FI group, with HR=1.54 (95% CI: 1.11 to 2.12). However, no significant difference in 28-day mortality was found between the middle-risk FI group and the low-risk FI group, with HR=1.48 (95% CI: 0.93 to 2.37) (Figure 6). In survival analyses by FI status, there was no statistically significant difference but a trend toward higher 28-day mortality in patients with FI *vs.* those without (HR=1.27, 95% CI: 0.96 to 1.67; log-rank *P*=0.092; Supplementary Figure S3). The effect direction was consistent with the NOFI strata: higher predicted FI risk and higher observed FI incidence were both associated with poorer 28-day survival, supporting internal consistency.

## Discussion

In this post hoc analysis of the NEED study, we evaluated that the performance of NOFI. Although NOFI demonstrates acceptable predictive performance, its ability to discriminate true negatives and accurately predict low-risk patients requires further improvement. Our study also indicates that critically ill patients at high risk of FI have a higher 28-day mortality rate compared to those at middle or low risk. Early identification of high-risk FI patients and the development and implementation of personalized nutritional and medical strategies could be beneficial for patients.

The FI predictive machine learning model developed by Raphaeli et al.^[^^10^^]^ may be cumbersome in clinical practice, and the cost of obtaining predictive factors may not be aligned with the prediction outcomes. The predictive models applied to clinical practice should not only consider their discrimination and calibration, but also the convenience and practicability are very crucial factors. Several clinical studies have developed prediction models for FI. Lu et al.^[^^8^^]^ constructed a model in a prospective cohort but without external validation. The models by Liu et al.^[^^7^^]^ and Hu et al.^[^^9^^]^ were derived from retrospective cohorts, limiting data quality. All three studies used relatively small samples, featured heterogeneous predictors, and their performance is uncertain due to small sample sizes and/or lower data quality. By contrast, Wang et al.’s^[^^6^^]^ NOFI model was built on a larger sample and underwent independent external validation in a prospective cohort. In addition, there are differences in the diagnostic criteria for FI, outcome occurrence intervals, and other definitions across these predictive models. Given the considerations regarding FI diagnostic standards and clinical practices, this study chose to perform independent external validation only for NOFI. Unfortunately, the performance of NOFI is not as good as imagined. Although it includes only three predictive factors, making it easy for clinical application, the NEED study was conducted before the 2019 ESPEN guidelines were published, so some patients did not follow the progressive feeding approach, which could have increased the incidence of FI. This also affects the FI prediction results, as all predictive factors were based on the patients’ initial status without considering the impact of the nutritional implementation process. This may be one reason why NOFI has relatively low specificity for predicting FI. Among the predictive factors, only the AGI grade is directly related to GI issues. However, one of the main limitations of the AGI grade is its subjectivity. There is a need to develop biomarkers related to GI function to optimize the model further. Future FI prediction should be individualized, but NOFI was designed for all critically ill patients who can initiate EEN.

This study has the following limitations: (1) Due to various limitations, it is not possible to compare different FI prediction models, and external validation was only performed for one model, which remains a limitation; (2) Although the NEED study largely reflects clinical practice in the Chinese mainland, the diverse clinical interventions across 97 ICUs may have had a potential impact on the outcomes; (3) In the NEED study, there were differences in nutritional intervention between the experimental group and the control group; Although this difference was not reflected in daily EN intake, it may still have a potential impact on the occurrence of FI.

Although NOFI demonstrated moderate performance in the NEED cohort, further studies are warranted to revalidate or optimize the model. So far, this is a highly reliable FI prediction model that has been verified many times and has clinical application value. Future optimization of NOFI should consider incorporating novel predictors – such as biomarkers and GI ultrasound metrics – to broaden predictor coverage and achieve more objective, higher-accuracy predictions. Future optimized or newly developed predictive models should take into account factors such as the convenience of clinical application, the ease of obtaining predictive factors, the cost of obtaining these factors, and the balance between these costs and the value of the outcomes, rather than being limited to the model’s performance solely from a statistical analysis perspective.

## Conclusions

In the secondary analysis of the NEED study, NOFI showed reasonable performance but still requires improvement in model performance. Patients at high risk for FI had a higher 28-day mortality compared to those at middle or low risk. However, no significant difference in 28-day mortality was found between patients at middle risk and those at low risk for FI.

## CRediT authorship contribution statement

**Youquan Wang:** Writing – review & editing, Writing – original draft, Methodology, Formal analysis, Data curation, Conceptualization. **Yanhua Li:** Writing – review & editing, Software, Data curation. **Hongxiang Li:** Writing – review & editing, Methodology. **Nan Li:** Writing – review & editing. **Xinyu Li:** Writing – review & editing. **Dongfang Lv:** Data curation. **Lingling Bao:** Data curation. **Xuewen Feng:** Data curation. **Lu Ke:** Writing – review & editing, Formal analysis, Conceptualization. **Dong Zhang:** Writing – review & editing, Formal analysis, Conceptualization.

## Appendix Supplementary materials

Image, application 1

## References

[1] Singer P., Blaser A.R., Berger M.M. (2023). ESPEN practical and partially revised guideline: clinical nutrition in the intensive care unit. Clin Nutr.

[2] Compher C., Bingham A.L., McCall M., Patel J., Rice T.W., Braunschweig C. (2022). Guidelines for the provision of nutrition support therapy in the adult critically ill patient: the American society for parenteral and enteral nutrition. JPEN J Parenter Enteral Nutr.

[3] Guan X., Chen D., Xu Y. (2024). Clinical practice guidelines for nutritional assessment and monitoring of adult ICU patients in China. J Intensive Med.

[4] Reintam Blaser A., Malbrain M.L., Starkopf J., Fruhwald S., Jakob S.M., De Waele J. (2012). Gastrointestinal function in intensive care patients: terminology, definitions and management. Recommendations of the ESICM working group on abdominal problems. Intensive Care Med.

[5] Reintam Blaser A., Poeze M., Malbrain M.L., Björck M., Oudemans-van Straaten H.M., Starkopf J. (2013). Gastrointestinal symptoms during the first week of intensive care are associated with poor outcome: a prospective multicentre study. Intensive Care Med.

[6] Wang Y., Li Y., Wang H., Li H., Li Y., Zhang L. (2023). Development and validation of a nomogram for predicting enteral feeding intolerance in critically ill patients (NOFI): mixed retrospective and prospective cohort study. Clin Nutr.

[7] Liu L., Li J., Hu L., Cai X., Li X., Bai Y. (2025). Development and validation of a prediction model for enteral feeding intolerance in critical ill patients: a retrospective cohort study. J Clin Nurs.

[8] Lu X.M., Jia D.S., Wang R., Yang Q., Jin S.S., Chen L. (2022). Development of a prediction model for enteral feeding intolerance in intensive care unit patients: a prospective cohort study. World J Gastrointest Surg.

[9] Hu K., Deng X.L., Han L., Xiang S., Xiong B., Pinhu L. (2022). Development and validation of a predictive model for feeding intolerance in intensive care unit patients with sepsis. Saudi J Gastroenterol.

[10] Raphaeli O., Statlender L., Hajaj C., Bendavid I., Goldstein A., Robinson E. (2023). Using machine-learning to assess the prognostic value of early enteral feeding intolerance in critically ill patients: a retrospective study. Nutrients.

[11] Ke L., Lin J., Doig G.S., van Zanten A.R.H., Wang Y., Xing J. (2022). Actively implementing an evidence-based feeding guideline for critically ill patients (NEED): a multicenter, cluster-randomized, controlled trial. Crit Care.

