# Supplementary material for: External validation of the feeding intolerance prediction model (NOFI) in critically ill patients: A post hoc analysis of a large-scale randomized controlled trial
Source: J Intensive Med. 2026 Feb 2;6(3):239–46. doi: 10.1016/j.jointm.2025.12.004 (PMC13184461; doi:10.1016/j.jointm.2025.12.004)
Supplement: Supplementary file 1 [file mmc1.docx]

**Table S1. The logistic regression analysis parameters of NOFI**

| Variable | β |
| --- | --- |
| Primary diagnosis |  |
| Respiratory | -0.789 |
| Circulatory | 0.882 |
| AGI grade | 1.6 |
| APACHE II score | 0.098 |
| Constant | -3.163 |

Abbreviation: APACHE II, Acute physiology and chronic health evaluation II; AGI, Acute gastrointestinal injury.

**Table S2. Comparison of different feeding intolerance prediction models**

| Author | Year | Patients | Independent external verification | Prospective cohort | Model parameters |
| --- | --- | --- | --- | --- | --- |
| Wang et al.(1) | 2023 | Critically ill patients | Yes | Yes | Yes |
| Liu et al.(2) | 2025 | Critically ill patients | Yes | No | Yes |
| Lu et al.(3) | 2022 | Critically ill patients | No | Yes | No |
| Hu et al.(4) | 2022 | Sepsis Patients | Yes | No | No |
| Raphaeli et al.(5) | 2023 | Critically ill patients | No | No | No |


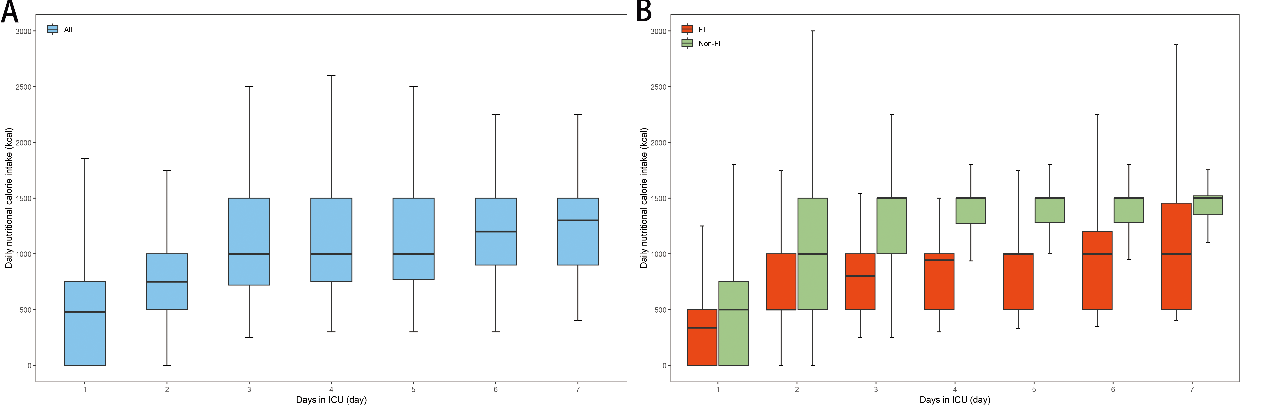


**Figure S1.** **Daily nutritional calorie intake for all patients (A), feeding intolerance (FI) patients and non-FI patients (B) during the 1-7 days after intensive care unit (ICU) admission.** Intake of calorie was calculated for all days during ICU admission where nutritional intake could be fully quantified. Horizontal lines in boxes represent medians; bottoms of boxes show 25th percentile and tops of boxes show 75th percentile. Ends of whiskers represent the upper adjacent value (ie, 75th percentile plus 1.5 times the IQR) and the lower adjacent value (ie, 25th percentile minus 1.5 times the IQR).


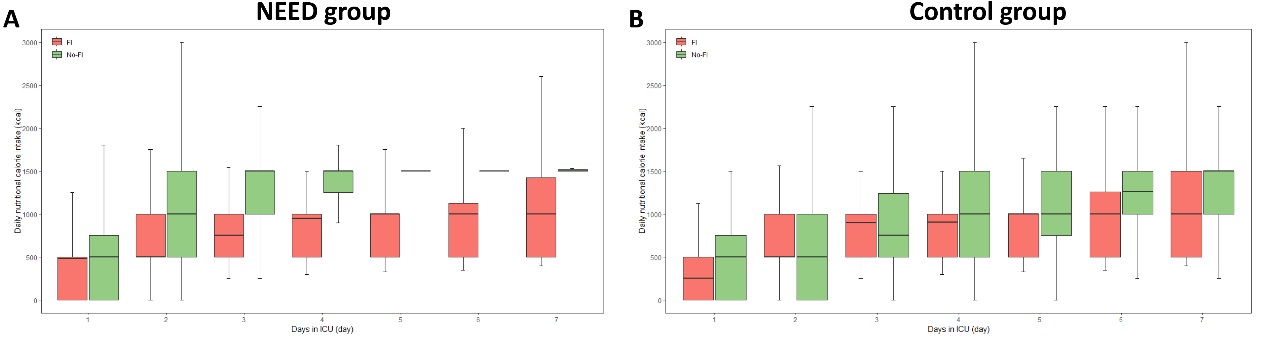


**Figure S2.** **Daily nutritional calorie intake for feeding intolerance (FI) patients and non-FI patients in the NEED group (A) and the control group (B) during the 1-7 days after intensive care unit (ICU) admission.** Intake of calorie was calculated for all days during ICU admission where nutritional intake could be fully quantified. Horizontal lines in boxes represent medians; bottoms of boxes show 25th percentile and tops of boxes show 75th percentile. Ends of whiskers represent the upper adjacent value (ie, 75th percentile plus 1.5 times the IQR) and the lower adjacent value (ie, 25th percentile minus 1.5 times the IQR).


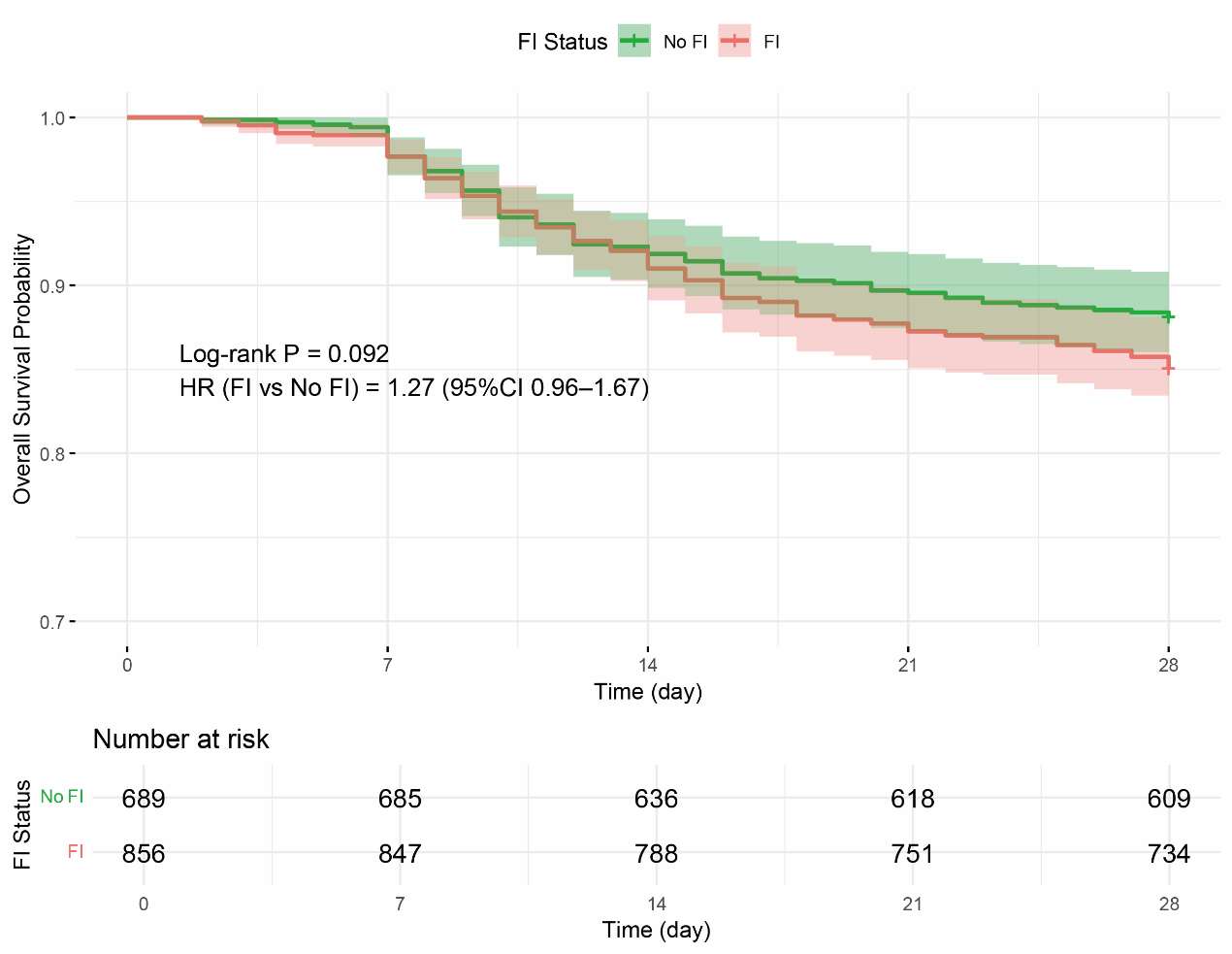


**Figure S3.** **The Cox regression survival curve between whether feeding intolerance occurs and the 28-day mortality.** Cox regression adjusted for APACHE II score, AGI grade, and MV. Time zero (t₀) was defined as ICU admission (day 0).

1. Wang Y, Li Y, Wang H, Li H, Li Y, Zhang L, et al. Development and validation of a nomogram for predicting enteral feeding intolerance in critically ill patients (NOFI): Mixed retrospective and prospective cohort study. Clin Nutr. 2023;42(12):2293-301.

2. Liu L, Li J, Hu L, Cai X, Li X, Bai Y. Development and Validation of a Prediction Model for Enteral Feeding Intolerance in Critical Ill Patients: A Retrospective Cohort Study. J Clin Nurs. 2025.

3. Lu XM, Jia DS, Wang R, Yang Q, Jin SS, Chen L. Development of a prediction model for enteral feeding intolerance in intensive care unit patients: A prospective cohort study. World J Gastrointest Surg. 2022;14(12):1363-74.

4. Hu K, Deng XL, Han L, Xiang S, Xiong B, Pinhu L. Development and validation of a predictive model for feeding intolerance in intensive care unit patients with sepsis. Saudi J Gastroenterol. 2022;28(1):32-8.

5. Raphaeli O, Statlender L, Hajaj C, Bendavid I, Goldstein A, Robinson E, et al. Using Machine-Learning to Assess the Prognostic Value of Early Enteral Feeding Intolerance in Critically Ill Patients: A Retrospective Study. Nutrients. 2023;15(12).
